# Somatosensory Induced Cerebellar Responses to Peripheral Nerve Stimulation: A Time and Time–Frequency EEG Study

**DOI:** 10.3390/brainsci16020132

**Published:** 2026-01-26

**Authors:** Anna Latorre, Kais Humaidan, Mauro Sanna, Maria Lucrezia Lavena, Anna Maria Contu, Maria Giuseppina Mele, Elias Paolo Casula, Lorenzo Rocchi

**Affiliations:** 1 Department of Clinical and Movement Neurosciences, UCL Queen Square Institute of Neurology, University College London, London WC1N 3BG, UK; a.latorre@ucl.ac.uk; 2 Department of Medical Sciences and Public Health, University of Cagliari, 09124 Cagliari, Italy; humaidankais@gmail.com (K.H.); m.sanna@protonmail.com (M.S.); malulavena@gmail.com (M.L.L.); giuseppinameleuni@gmail.com (M.G.M.); 3 Department of Neurology and Stroke Unit, Mater Olbia Hospital, 07026 Olbia, Italy; annamariacontu0@gmail.com; 4 Department of System Medicine, “Tor Vergata” University of Rome, Via Montpellier 1, 00133 Rome, Italy; elias.casula@gmail.com

**Keywords:** cerebellum, electroencephalography, evoked potentials, somatosensory evoked potentials, peripheral nerve stimulation, neurophysiology

## Abstract

**Background/Objectives:** The cerebellum plays a central role in sensorimotor integration and temporal processing, yet its direct electrophysiological investigation in humans remains challenging, and cerebellar contributions to somatosensory responses remain poorly defined. This study aimed to determine whether cerebellar responses to peripheral nerve stimulation can be detected using scalp EEG and whether time–frequency analysis provides advantages over time-domain approaches. **Methods:** Scalp EEG was recorded during electrical stimulation of the median nerve and tibial nerve in 16 healthy participants. Electrode montages included posterior fossa placements targeting cerebellar activity, together with standard cortical and subcortical derivations. Data were analyzed in the time domain using evoked potentials and channel comparisons, including bipolar cerebellar derivations, and in the time–frequency domain using spectral power analysis. **Results:** Time-domain analyses revealed early and intermediate latency components following both upper- and lower-limb stimulation; however, these responses showed limited spatial specificity and were strongly influenced by reference effects and subcortical contamination. In contrast, time–frequency analysis consistently revealed sustained increases in oscillatory power in cerebellar channels. Power increases emerged approximately 50 ms after stimulation and persisted beyond 300 ms, peaking around ~20 Hz for upper-limb stimulation and ~10 Hz for lower-limb stimulation, with evidence of side specificity. **Conclusions:** Non-invasive EEG can detect cerebellar responses to peripheral nerve stimulation, particularly in the time–frequency domain. Oscillatory dynamics provide a more robust marker of cerebellar involvement than time-locked responses and may complement conventional somatosensory evoked potentials in studies of cerebellar physiology and spinocerebellar pathway integrity.

## 1. Introduction

The cerebellum is a central structure in human motor control, playing a fundamental role in the coordination, timing, and precision of voluntary movements, as well as in the maintenance of posture and balance [1,2]. These functions arise from the cerebellum’s capacity to integrate multisensory inputs with descending motor commands, allowing continuous updating and fine-tuning of motor output [3,4]. In addition to its classical motor functions, the cerebellum contributes to motor learning [5,6], error-based adaptation, pain processing [7] and higher-order cognitive operations, including temporal prediction and sensorimotor integration [8]. Despite its functional relevance, the direct electrophysiological investigation of cerebellar activity in humans remains technically challenging. Several anatomical and physiological factors limit the detectability of cerebellar signals at the scalp, including the relatively large distance between the cerebellar cortex and recording electrodes, the fine folding of cerebellar folia, the predominantly closed-field geometry of Purkinje cells, low levels of neuronal synchrony, and the prominence of high-frequency activity [9,10,11,12,13]. As a result, cerebellar function in intact humans has traditionally been studied using indirect methods, such as eyeblink conditioning [14,15] cerebellar–brain inhibition [16,17,18], and cerebellar evoked responses elicited by transcranial magnetic stimulation [19,20,21].

Recent methodological advances have renewed interest in the possibility of directly recording cerebellar activity non-invasively using scalp EEG. Signals obtained from electrodes positioned over the posterior fossa, commonly referred to as electrocerebellogram (ECeG) [22,23], are thought to reflect cerebellar local field potentials. Accumulating evidence indicates that ECeG activity is modulated by vestibular, optokinetic, auditory, and visual stimulation, as well as by eye movements and motor tasks, suggesting a predominant contribution from the vestibulo-cerebellum [14,22,24,25,26,27]. Moreover, scalp-recorded cerebellar activity has been linked not only to postural control and gait disturbances in Parkinson’s disease, particularly through oscillatory activity in the theta band, but also to upper-limb motor control and tremor modulation, further supporting the functional relevance of these signals [28,29,30].

Among its multiple roles, the cerebellum is critically involved in somatosensory processing, especially with regard to proprioceptive information. Peripheral somatosensory inputs reach the cerebellum through anatomically distinct ascending pathways, including the dorsal and ventral spinocerebellar tracts for the lower limbs and the cuneocerebellar and rostral spinocerebellar tracts for the upper limbs [31,32]. These pathways primarily convey unconscious proprioceptive information and are largely segregated from the dorsal column–medial lemniscus system, which transmits conscious tactile and proprioceptive signals to the cerebral cortex.

Somatosensory evoked potentials (SEPs) represent a well-established technique for investigating somatosensory pathways in humans [33]. SEPs consist of time-locked electrophysiological responses elicited by electrical stimulation of peripheral nerves and reflect the sequential activation of neural generators along the dorsal column–medial lemniscus pathway, from peripheral afferents to the primary somatosensory cortex [33,34]. In clinical practice, SEPs are widely used to assess the functional integrity of large-diameter afferent fibers [34]. However, because SEPs primarily probe lemniscal pathways, they provide limited information about spinocerebellar afferent processing and cerebellar involvement in somatosensory integration.

Given the anatomical organization of somatosensory pathways, peripheral nerve stimulation should activate both lemniscal and spinocerebellar afferents, making the elicitation of cerebellar responses physiologically plausible. Early animal studies demonstrated cerebellar field potentials evoked by peripheral stimulation that selectively reflected the integrity of ventral spinal pathways [35]. Subsequent intraoperative studies in humans reported cerebellar evoked responses following tibial nerve stimulation, with waveforms distinct from cortical SEPs, supporting a non-lemniscal, likely spinocerebellar origin [36]. Comparable responses were also recorded at the C2 spinal level, further supporting ventral transmission [37,38]. Further support was later provided by magnetoencephalography studies demonstrating cerebellar responses preceding cortical activation after median nerve stimulation, with oscillatory dynamics consistent with cerebellar involvement in sensory processing and temporal prediction [39,40].

Despite these observations, the literature on cerebellar responses to somatosensory input remains sparse and methodologically heterogeneous. Many studies relied on invasive recordings, limited spatial coverage, or analytical approaches optimized for neocortical activity. Importantly, cerebellar responses may be weakly phase-locked and dominated by high-frequency components, making them difficult to detect using conventional averaging techniques typically employed for SEPs [10]. Consequently, cerebellar somatosensory activity has rarely been systematically investigated using combined time-domain and time–frequency analyses in non-invasive scalp recordings.

The present study aims to determine whether reproducible cerebellar responses (surface cerebellar evoked potentials—sCEP) to peripheral nerve stimulation can be recorded non-invasively using scalp electrodes positioned over the posterior fossa. By systematically comparing electrophysiological signals recorded at different stages of the somatosensory pathway with those obtained from cerebellar electrodes, and by analyzing both time-domain and time–frequency features, we seek to identify responses of cerebellar origin. Demonstrating the feasibility and specificity of such recordings would provide new insights into cerebellar somatosensory processing and open novel perspectives for investigating spinocerebellar function in both physiological and pathological conditions.

## 2. Materials and Methods

### 2.1. Participants

Sixteen right-handed healthy volunteers [41] (mean age 30.4 ± 6.5 years; eight females) participated in the study. None had a history of neurological or psychiatric disorders, and none were taking medications acting on the central nervous system. All procedures were conducted in accordance with the Declaration of Helsinki and approved by the local review board (ID PG/2018/8829). Written informed consent was obtained from all participants before the experimental session.

### 2.2. Experimental Design and Data Recording

Subjects lay supine on a couch with a pillow under the head to maximize comfort and minimize tonic contraction of the neck muscles. Two recording blocks—upper-limb SEPs and lower-limb SEPs—were performed in a single experimental session, with block order randomized. In both blocks, signals were recorded using surface Ag/AgCl cup electrodes, and electrode impedance was kept below 5 kΩ.

Stimulation was delivered with a constant-current electrical stimulator (Digitimer DS7A, Digitimer Ltd., Welwyn Garden City, UK). A total of 1000 monophasic square-wave pulses (200 μs duration) were delivered at 1 ± 0.1 Hz. Stimulation intensity was set at 1.5 times the motor threshold, defined as the minimum intensity required to elicit a compound muscle action potential (CMAP) of 50–100 μV in the abductor pollicis brevis (for median nerve SEPs) or the abductor hallucis (for tibial nerve SEPs). CMAPs were recorded both to determine the stimulation threshold and to monitor the stability of nerve activation throughout each block.

SEPs were recorded from −500 ms before the pulse to 400 ms after it, with a band-pass filter of 2–2000 Hz and a gain of 100,000×. CMAPs were recorded with a band-pass filter of 5–2000 Hz and a gain of 1000× using bipolar belly–tendon electrodes. All signals were acquired using a Digitimer D360 amplifier (Digitimer Ltd., Welwyn Garden City, UK), digitized with a CED1401 A/D system at 10 kHz, and displayed via Signal software version 7.06 (Cambridge Electronic Design Ltd., Cambridge, UK).

In the upper-limb SEP block, the right median nerve was stimulated at the wrist, with the cathode oriented proximally. The recording montage included electrodes at CP3, Fz, IpsS (ipsilateral shoulder), C5s (spinous process of the fifth cervical vertebra), Jn (jugular notch), CB1 and CB2 (left and right cerebellar hemispheres), and SP2 (right splenius muscle), positioned according to the international 10–20 system [42]. Cerebellar electrodes CB1 and CB2 were placed following prior evidence that this montage reliably captures cerebellar activity [22,30].

**Figure 1:**
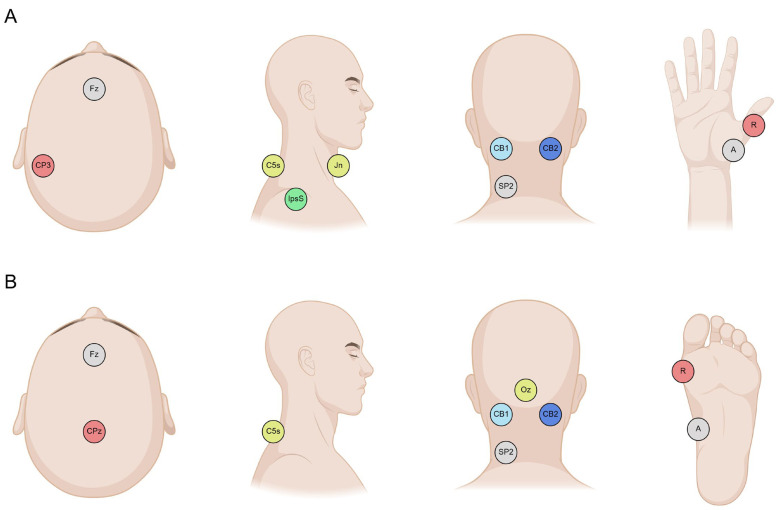
Approximate placement of recording electrodes on the scalp for upper-limb (**A**) and lower-limb (**B**) SEPs. Electrode nomenclature follows the 10–20 system except for the C5s (spinous process of the fifth cervical vertebra), IpsS (ipsilateral shoulder), Jn (jugular notch), CB1 and CB2 (cerebellar electrodes), and SP2 (right splenium muscles). For electromyography channels, ‘A’ indicates the active electrode, placed on the muscle belly of interest, while ‘R’ indicates the reference electrode, placed on the tendon. See text for details.

In the lower-limb SEP block, the right tibial nerve was stimulated at the ankle with the cathode proximally oriented. The recording montage consisted of CPz, Fz, C5s, Oz, CB1, CB2, and SP2. Electrode placement is shown in Figure 1. Channels were grouped as described in Table 1 to provide a comprehensive view of cortical and subcortical SEPs commonly used in clinical practice and to enable comparison with putative cerebellar SEPs, as detailed in the following sections.

A common Fz reference was used for most recording derivations to maximize the comparability of signals across channels. Electrode Fz was selected because it is routinely employed as an active recording site for bulbar SEP components, such as P14 following median nerve stimulation and P31 following tibial nerve stimulation, in standard clinical practice [33]. This choice, however, introduces a potential methodological issue when performing pairwise comparisons between derivations in which Fz serves either as the active electrode or as the reference, as this configuration may result in an instrumental phase reversal unrelated to underlying neurophysiological differences.

To account for this, when comparing channels with opposite polarity conventions involving Fz, the corresponding signals were polarity-inverted prior to statistical analysis, ensuring that observed differences reflected genuine physiological features rather than reference-related artifacts.

### 2.3. Data Analysis and Statistics

Signal analysis and statistical testing were performed in MATLAB (version 2020a; MathWorks Inc., Natick, MA, USA). Electrical stimulation artifacts were first removed by excising the signal from −2 to +2 ms around each stimulus pulse, with the missing data reconstructed using cubic interpolation. A fourth-order band-stop filter (48–52 Hz) was applied to suppress line noise. In subjects in whom it was present, electrocardiographic artifact was removed using the Cardiac Artifact Removal toolbox [43]. Signal epochs were baseline-corrected using the −50 to −5 ms interval, and trials with amplitudes exceeding 100 μV were rejected.

Given that afferent somatosensory projections to the cerebellum are predominantly unilateral, the CB2–Fz derivation was selected as the main channel of interest. For upper-limb SEPs, pairwise comparisons were performed between CB2–Fz and CP3–Fz, Fz–IpsS, C5s–Jn, and CB1–Fz. For lower-limb SEPs, signals from CB2–Fz were compared with those recorded from CPz–Fz, Fz–C5s, Oz–Fz, and CB1–Fz. Statistical comparisons were conducted over the entire post-stimulus time window (0–400 ms) using a non-parametric cluster-based permutation approach. Group-mean differences were tested against a null distribution generated by randomly shuffling condition labels across 1000 permutations. For each permutation, pointwise differences were z-scored and thresholded at *p* < 0.05 (two-tailed), and clusters of contiguous supra-threshold samples were identified using MATLAB’s bwconncomp function. The largest cluster from each permutation was used to construct the null distribution of cluster sizes. Observed clusters were evaluated against this distribution, and clusters smaller than the 95th percentile were discarded. Final *p*-values were derived from the standard normal cumulative distribution [44]. This procedure controls for multiple comparisons without relying on parametric assumptions.

Based on the hypothesis that putative cerebellar responses would manifest as spatially restricted, closely spaced fields, signals from the CB1–Fz derivation were subtracted from those recorded at CB2–Fz for both upper- and lower-limb SEPs, yielding a CB2–CB1 derivation. In this case, statistical testing followed the same permutation framework, with the artificial signal in the 0–400 ms interval compared against its own baseline average (−100 to −5 ms).

Time–frequency representations were obtained by computing the analytic signal via Morlet wavelet decomposition (1–50 Hz; 4–10 cycles, increasing logarithmically across frequencies) [45]. Power changes were expressed as ratios of post-stimulus activity (0–400 ms) relative to a frequency-specific baseline (−500 to −50 ms). Statistical analysis of time–frequency data followed the same cluster-based permutation procedure described above, using the full time × frequency matrices as input. Here, we focused on positive clusters only, i.e., where power in CB2 was larger compared to the channel being compared.

## 3. Results

All subjects successfully completed the experimental session. Stimulation intensity was 7.24 ± 2.93 mA for median nerve SEPs and 9.23 ± 1.85 mA for tibial nerve SEPs. The number of retained trials was 924 ± 13 for upper-limb SEPs and 905 ± 17 for lower-limb SEPs. Electrode SP2 did not show EMG activity, which suggests that the signals recorded at CB2 were not contaminated by EMG. All figures show negative potentials upwards and positive potentials downwards.

### 3.1. Upper-Limb SEPs

**Figure 2:**
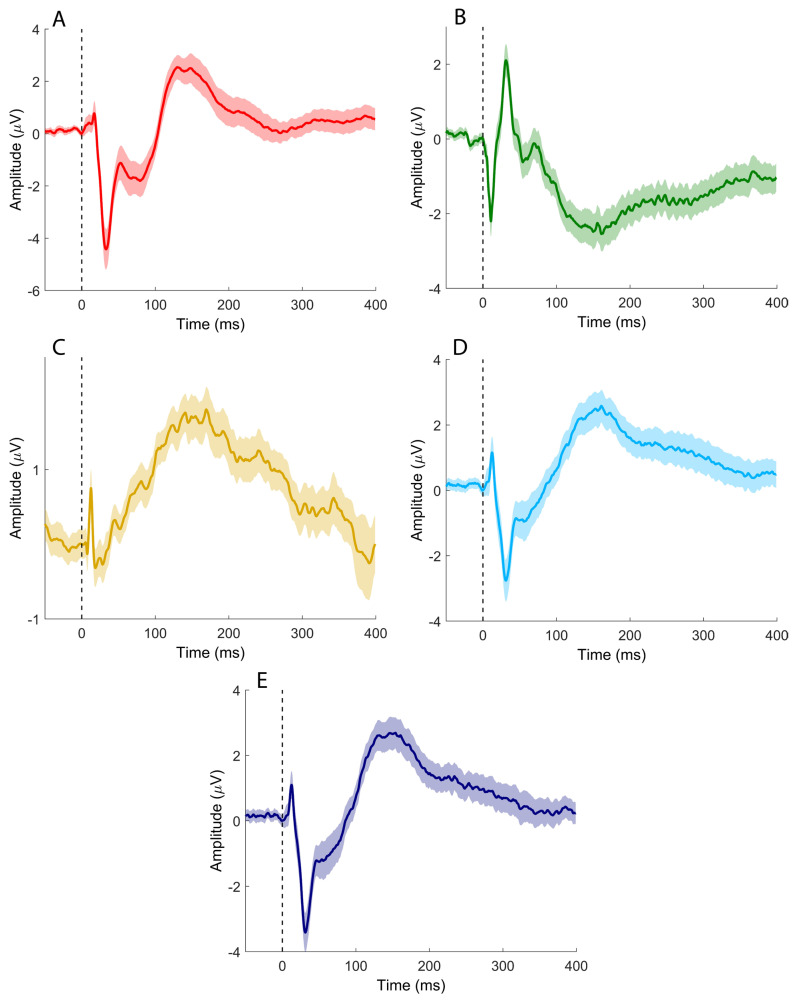
Time-domain signals recorded after median nerve stimulation. (**A**) CP3-Fz; (**B**) Fz-IpsS; (**C**) C5s-Jn; (**D**) CB1-Fz; (**E**) CB2-Fz. Colored, solid lines represent signals averaged at the group level. Shaded areas depict the standard error of the mean. Black vertical dashed lines indicate the timing of the electrical pulse.

Median nerve stimulation elicited the SEP components commonly observed in clinical practice (Figure 2A–C), together with putative sCEPs recorded over the left and right cerebellar hemispheres (Figure 2D and Figure 2E, respectively). sCEPs were mainly characterized by an early negative–positive deflection (<50 ms), followed by a prominent, slower negative peak occurring between approximately 100 and 200 ms.

**Figure 3:**
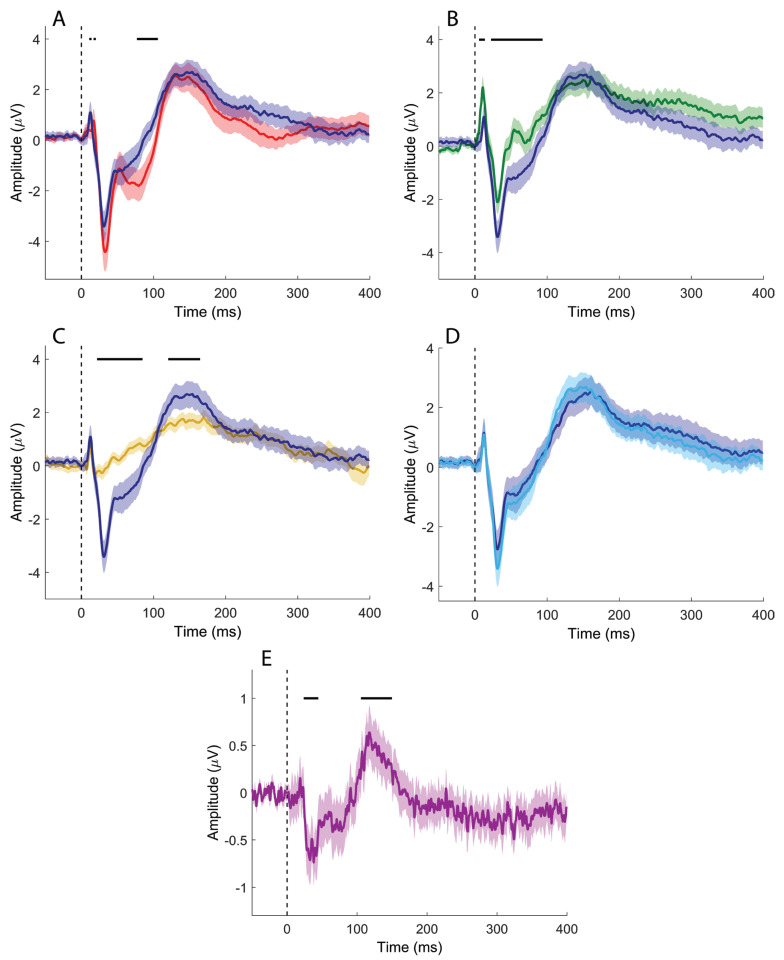
Time-domain signal comparisons (median nerve stimulation). (**A**) CB2-Fz (dark blue) vs. CP3-Fz (red); (**B**) CB2-Fz (dark blue) vs. C5s-Jn flipped (green); (**C**) CB2-Fz (dark blue) vs. C5s-Jn (yellow); (**D**) CB2-Fz (dark blue) vs. CB1-Fz (light blue); (**E**) CB2-CB1 (purple) vs. baseline. Colored, solid lines represent signals averaged at the group level. Shaded areas depict the standard error of the mean. Black vertical dashed lines indicate the timing of the electrical pulse. Continuous black horizontal lines indicate significant differences.

When the channel of interest (CB2–Fz) was compared with the derivation used to record the N20/P25 complex (CP3–Fz; Figure 3A), significant differences emerged at 11.5–14.7 ms, 17.6–21.2 ms, and 77.4–106.2 ms. In the comparison with the P14/N18 components recorded from the Fz–IpsS derivation (Figure 3B), significant clusters were observed at 6.6–13.7 ms and 22.3–93.8 ms. In this case, the Fz–IpsS signal was inverted to avoid instrumental phase reversal due to the use of the same electrode as either active or reference across derivations.

Comparison with the derivation used to record the N13 (C5s–Jn; Figure 3C) revealed significant clusters at 21.6–44.6 ms and 105.5–149.8 ms. No significant differences were detected when directly comparing CB2–Fz and CB1–Fz signals (Figure 3D).

However, when the CB2–CB1 derivation was compared with its baseline (Figure 3E), significant clusters emerged at 23.7–44.6 ms and 105.5–149.8 ms, indicating lateralized cerebellar activity not apparent in the direct channel-to-channel comparison.

**Figure 4:**
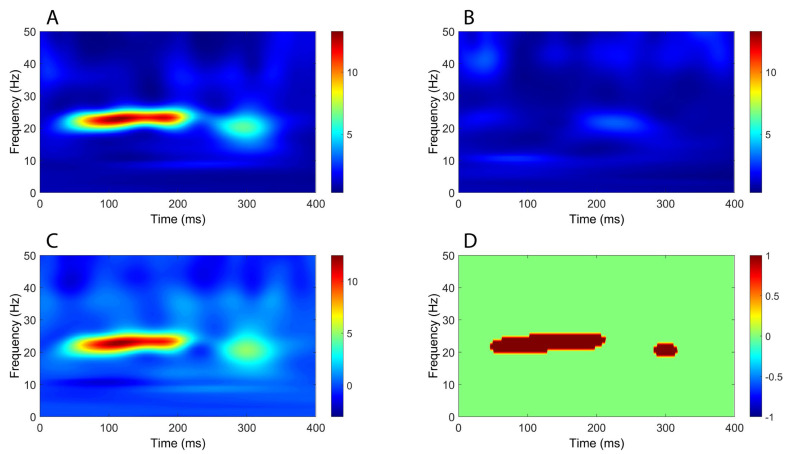
Comparison of time–frequency domain responses between CB2-Fz and CP3-Fz channels (median nerve stimulation). (**A**) CB2-Fz; (**B**) CP3-Fz; (**C**) CB2-Fz minus CP3-Fz; (**D**) map of significant differences between channels. (**A**–**C**) indicate normalized power ratio, while (**D**) depicts z scores.

**Figure 5:**
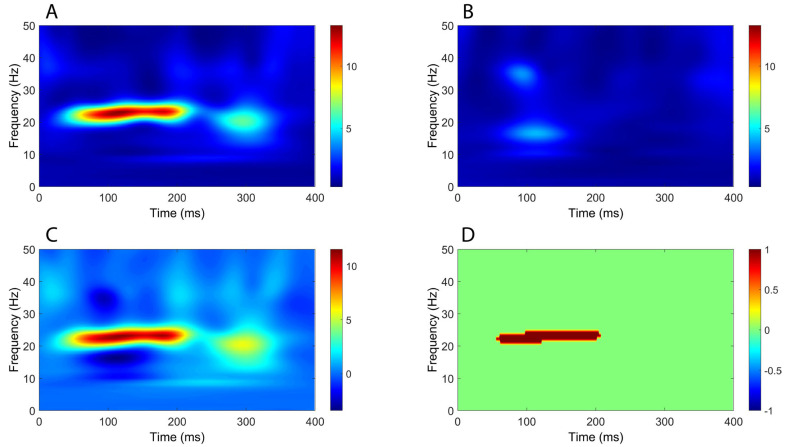
Comparison of time–frequency domain responses between CB2-Fz and Fz-IpsS channels (median nerve stimulation). (**A**) CB2-Fz; (**B**) Fz-IpsS; (**C**) CB2-Fz minus Fz-IpsS; (**D**) map of significant differences between channels. (**A**–**C**) indicate normalized power ratio, while (**D**) depicts z scores.

**Figure 6:**
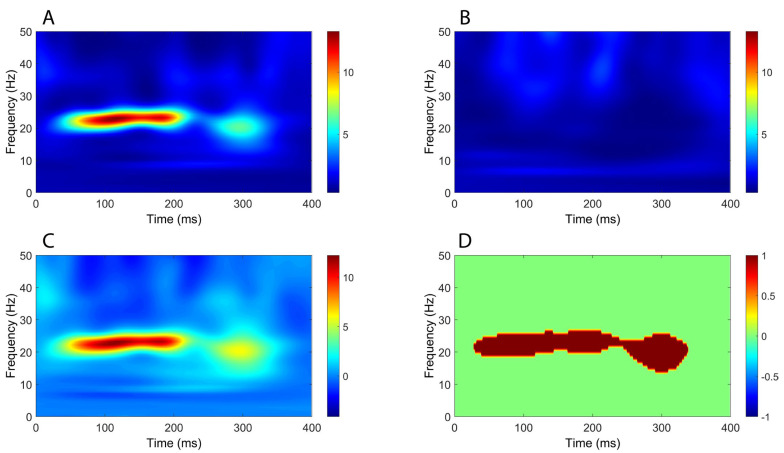
Comparison of time–frequency domain responses between CB2-Fz and C5s-Jn channels (median nerve stimulation). (**A**) CB2-Fz; (**B**) C5s-Jn; (**C**) CB2-Fz minus C5s-Jn; (**D**) map of significant differences between channels. (**A**–**C**) indicate normalized power ratio, while (**D**) depicts z scores.

**Figure 7:**
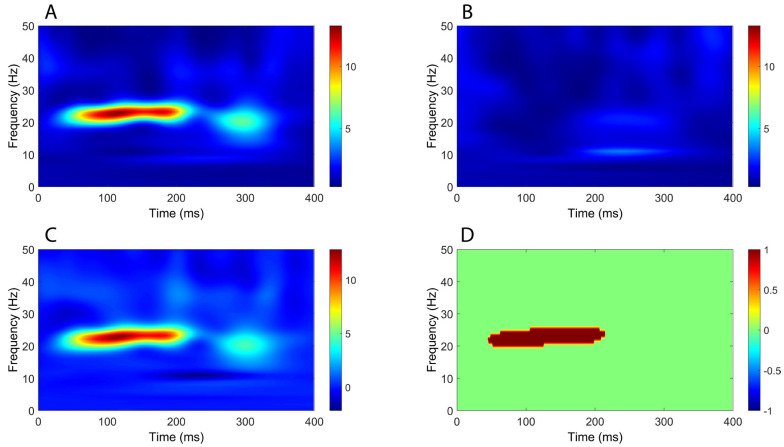
Comparison of time–frequency domain responses between CB2-Fz and CB1-Fz channels (median nerve stimulation). (**A**) CB2-Fz; (**B**) CB1-Fz; (**C**) CB2-Fz minus CB1-Fz; (**D**) map of significant differences between channels. (**A**–**C**) indicate normalized power ratio, while (**D**) depicts z scores.

Time–frequency analysis revealed a significant increase in oscillatory power mostly within the beta band (around 20 Hz), extending from around 50 ms to beyond 300 ms, in all comparisons between CB2–Fz and the other derivations (Figure 4, Figure 5, Figure 6 and Figure 7).

### 3.2. Lower-Limb SEPs

**Figure 8:**
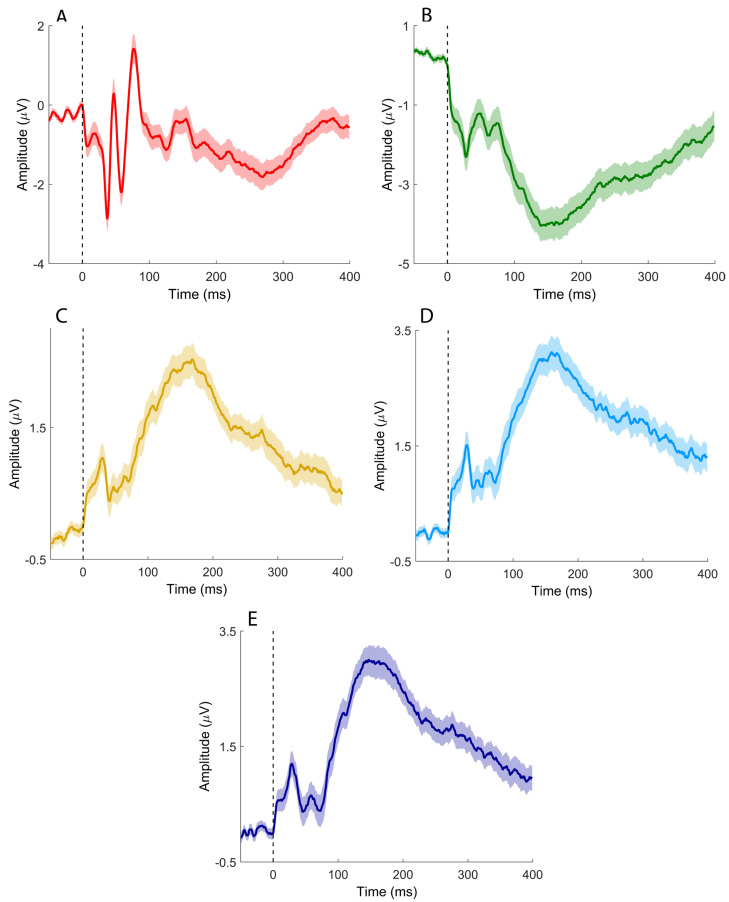
Time-domain signals recorded after tibial nerve stimulation. (**A**) CPz-Fz; (**B**) Fz-C5s; (**C**) Oz-Fz; (**D**) CB1-Fz; (**E**) CB2-Fz. Colored, solid lines represent signals averaged at the group level. Shaded areas depict the standard error of the mean. Black vertical dashed lines indicate the timing of the electrical pulse.

Tibial nerve stimulation elicited early SEP components typically observed in clinical recordings, including the P40 (Figure 8A) and P31 (Figure 8B). Recordings from cerebellar electrodes CB1 and CB2 (Figure 8D,E) were characterized by two prominent negative deflections: an early component around 30 ms and a later component between approximately 100 and 200 ms.

**Figure 9:**
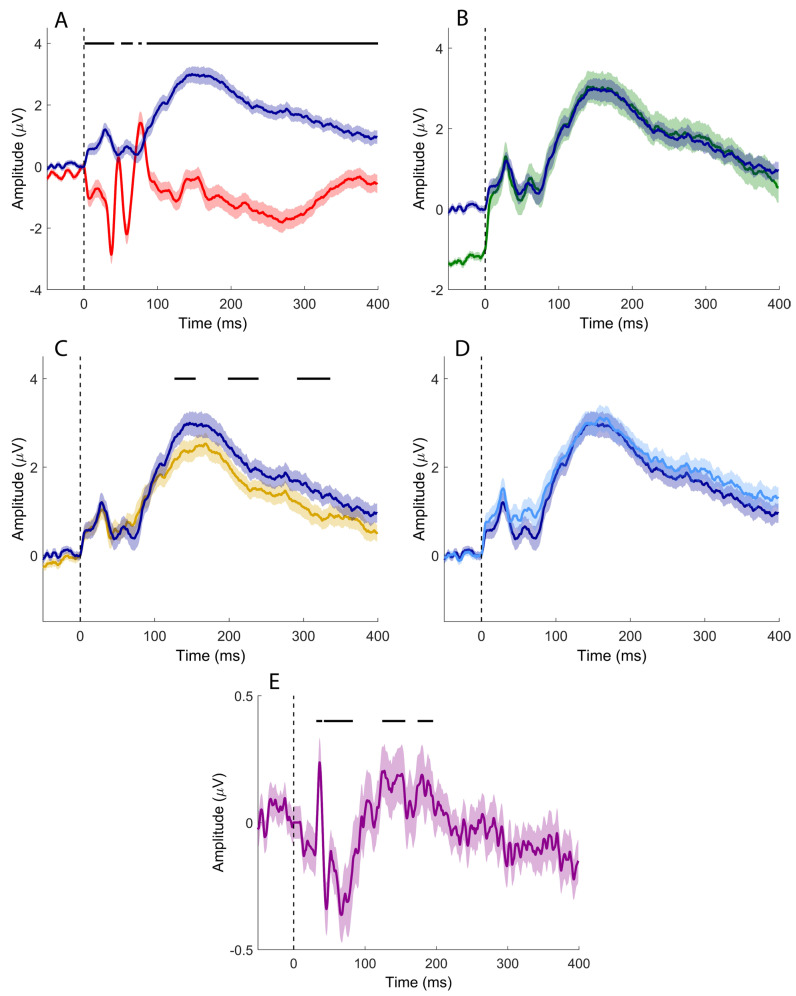
Time-domain signal comparisons (tibial nerve stimulation). (**A**) CB2-Fz (dark blue) vs. CPz-Fz (red); (**B**) CB2-Fz (dark blue) vs. Fz-C5s flipped (green); (**C**) CB2-Fz (dark blue) vs. Oz-Fz (yellow); (**D**) CB2-Fz (dark blue) vs. CB1-Fz (light blue); (**E**) CB2-CB1 (purple) vs. baseline. Colored, solid lines represent signals averaged at the group level. Shaded areas depict the standard error of the mean. Black vertical dashed lines indicate the timing of the electrical pulse. Continuous black horizontal lines indicate significant differences.

Comparison of the channel of interest (CB2–Fz) with the CPz–Fz derivation, used to record the P40, disclosed significant differences across most of the analyzed time window (2.1–41.1 ms, 50.4–66.4 ms, 73.8–78.6 ms, and 85.2–400 ms; Figure 9A), indicating limited temporal overlap between these signals. In contrast, no significant differences were observed when comparing CB2–Fz with the Fz–C5s derivation used to record the P31 (Figure 9B). Some significant clusters were detected in the comparison between CB2–Fz and Oz–Fz (126.6–155.2 ms, 198.4–239.6 ms, and 291.3–336 ms; Figure 9C).

As for upper-limb SEPs, the Fz–C5s derivation was inverted to avoid instrumental phase reversal in the comparison with CB2–Fz. However, the limited extent of significant differences, together with their absence in the comparison with Fz–C5s, suggests substantial contamination of putative cerebellar SEPs elicited by tibial nerve stimulation by sources within the posterior fossa.

No clear lateralization emerged from the direct comparison between CB2–Fz and CB1–Fz signals (Figure 9D). Nevertheless, comparison of the CB2–CB1 derivation with its baseline revealed significant clusters at 31.6–38.9 ms, 42.4–83.0 ms, 124.2–156.4 ms, and 173.7–185.5 ms, indicating side-specific cerebellar activity not evident in the raw channel comparison (Figure 9E).

**Figure 10:**
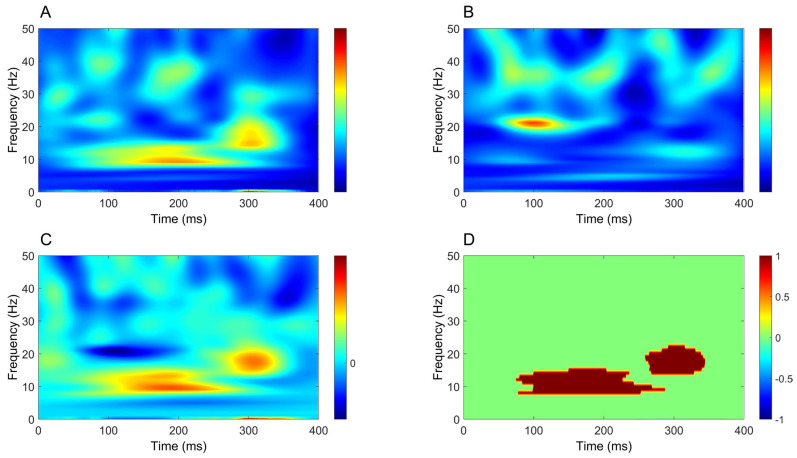
Comparison of time–frequency domain responses between CB2-Fz and CPz-Fz channels (tibial nerve stimulation). (**A**) CB2-Fz; (**B**) CPz-Fz; (**C**) CB2-Fz minus CPz-Fz; (**D**) map of significant differences between channels. (**A**–**C**) indicate normalized power ratio, while (**D**) depicts z scores.

**Figure 11:**
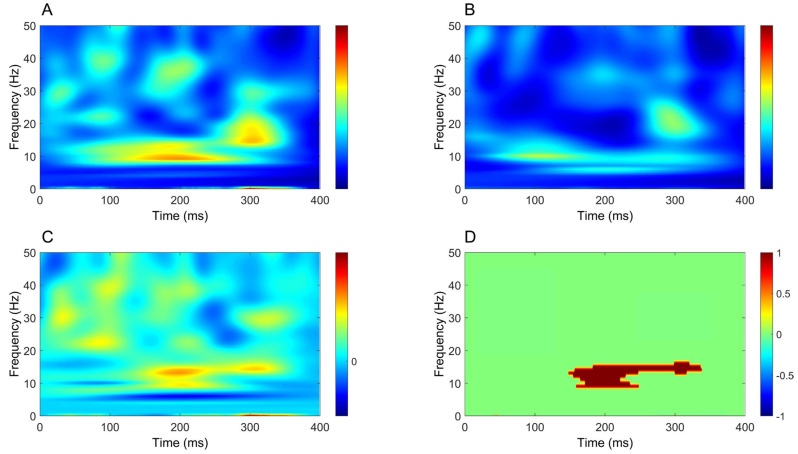
Comparison of time–frequency domain responses between CB2-Fz and Fz-C5s channels (tibial nerve stimulation). (**A**) CB2-Fz; (**B**) Fz-C5s; (**C**) CB2-Fz minus Fz-C5s; (**D**) map of significant differences between channels. (**A**–**C**) indicate normalized power ratio, while (**D**) depicts z scores.

**Figure 12:**
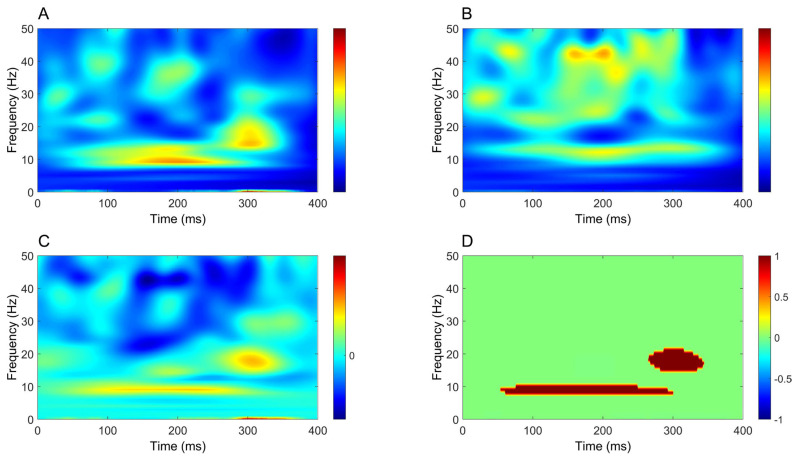
Comparison of time–frequency domain responses between CB2-Fz and Oz-Fz channels (tibial nerve stimulation). (**A**) CB2-Fz; (**B**) Oz-Fz; (**C**) CB2-Fz minus Oz-Fz; (**D**) map of significant differences between channels. (**A**–**C**) indicate normalized power ratio, while (**D**) depicts z scores.

**Figure 13:**
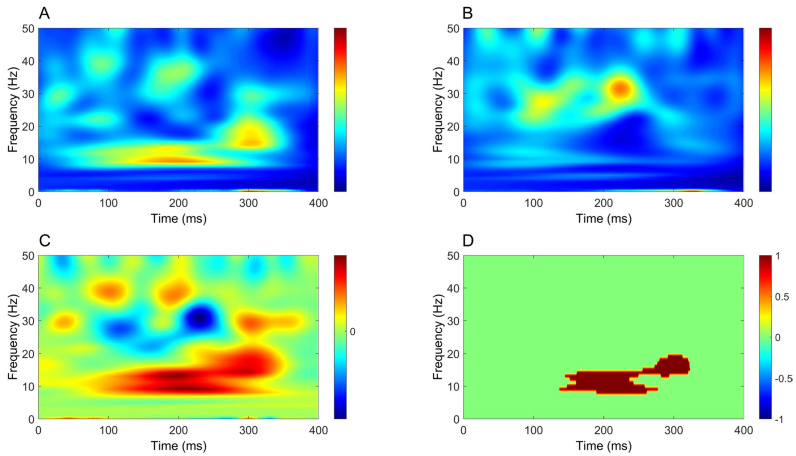
Comparison of time–frequency domain responses between CB2-Fz and CB1-Fz channels (tibial nerve stimulation). (**A**) CB2-Fz; (**B**) CB1-Fz; (**C**) CB2-Fz minus CB1-Fz; (**D**) map of significant differences between channels. (**A**–**C**) indicate normalized power ratio, while (**D**) depicts z scores.

Finally, similar to upper-limb stimulation, time–frequency analysis demonstrated a significant increase in power extending from approximately 50 ms to beyond 300 ms in all comparisons between CB2–Fz and the other derivations (Figure 10, Figure 11, Figure 12 and Figure 13), but this time the power change was mostly around 10 Hz.

## 4. Discussion

In this study, we examined whether reproducible cerebellar responses to peripheral nerve stimulation can be recorded non-invasively using scalp EEG. Median and tibial nerve stimulation elicited sCEPs characterized by an early negative component in the ~20–40 ms range and a later negative deflection peaking between approximately 100 and 200 ms. Time-domain analyses revealed several statistically significant differences between cerebellar and non-cerebellar derivations; however, these effects showed limited spatial specificity and were largely dominated by the common reference at Fz. In contrast, subtraction of signals from homologous cerebellar electrodes (CB2–CB1) revealed lateralized activity not evident in direct channel comparisons. In the time–frequency domain, peripheral nerve stimulation consistently induced sustained increases in oscillatory power, mainly in the alpha–beta range, extending from approximately 50 ms to beyond 300 ms, providing convergent evidence for cerebellar involvement in somatosensory processing.

### 4.1. Time-Domain Analysis of Cerebellar Responses

For upper-limb stimulation, time-domain analysis revealed several clusters of statistical significance; however, these effects lacked clear spatial specificity attributable to cerebellar generators. Comparisons based on the channel of interest (CB2–Fz) were strongly influenced by the common reference at Fz. Differences between CB2–Fz and CP3–Fz were primarily driven by the cortical SEP complex (N20/P25) (Figure 3A), with additional contamination from subcortical activity, most notably the cervical N13, and a further difference emerging just before 100 ms of uncertain origin. Comparisons with Fz–IpsS highlighted differences associated with the P14/N18 complex; in this case, the derivation was reversed to avoid spurious effects due to instrumental phase reversal, as Fz served as the reference for CB2–Fz and as the active electrode for Fz–IpsS (Figure 3B). Although both early- and intermediate-latency differences were observed, the absence of early differences in the comparison between CB2–Fz and C5s–Jn suggests that early cerebellar responses are likely dominated by subcortical contamination rather than reflecting genuine cerebellar generators (Figure 3C). Differences observed at intermediate latencies, spanning approximately 20–100 ms across all comparisons and extending up to 100–200 ms in the comparison with the cervical N13 channel, may reflect genuine cerebellar activity, but this interpretation remains uncertain given the lack of side specificity in the direct comparison between CB2–Fz and CB1–Fz (Figure 3D). A possible indication of lateralized cerebellar involvement emerges only when considering the bipolar CB2–CB1 derivation, where significant deviations from baseline suggest spatially restricted activity that becomes detectable only with near-field analysis (Figure 3E).

Findings from tibial nerve stimulation largely mirrored those observed for the upper limb. In the time domain, CB2–Fz showed little temporal overlap with the P40 channel (Figure 9A), indicating that cerebellar recordings were not driven by the main cortical SEP components. However, comparisons with other derivations revealed only modest differences (Figure 9B,C), largely attributable to widespread contamination from posterior fossa and subcortical sources. Classical tibial SEP components (P40, N45, N60, P100) were not clearly identifiable in cerebellar channels, and no early cerebellar-specific responses emerged. As in the upper-limb condition, direct comparison between CB2–Fz and CB1–Fz did not reveal side-specific differences, whereas the bipolar CB2–CB1 derivation disclosed significant deviations from baseline, suggesting the presence of a spatially restricted near-field component (Figure 9E).

These observations can be interpreted in light of earlier experimental and clinical studies. In animal models, direct sciatic nerve stimulation elicited a reproducible cerebellar evoked potential recorded over the paramedian lobule and transmitted predominantly via ventral spinal pathways [35]. Building on this work, Hurlbert and coworkers reported a homologous response in humans following tibial nerve stimulation, characterized by an early negative peak around 30–35 ms, followed by a positive deflection and a subsequent negative component, independent of classical SEPs [36]. The temporal structure of the residual CB2–CB1 response observed here during lower-limb stimulation is consistent with this sequence, although differences in recording modality and analysis preclude direct attribution to a common generator. Non-invasive magnetoencephalography studies further support cerebellar involvement: cerebellar responses following median nerve stimulation have been reported at latencies preceding cortical activation [39], and subsequent work has described a sequence of cerebellar activations spanning 15–70 ms, attributed to successive mossy fiber-, parallel fiber-, and climbing fiber-mediated Purkinje cell activity [46]. More recently, direct recordings from the human cerebellar surface demonstrated somatosensory evoked field potentials with short onset latencies, albeit in a limited subset of patients, underscoring the spatially restricted and condition-dependent nature of cerebellar responses [47].

Taken together, these comparisons suggest that the sCEPs observed here in the time domain are real but difficult to isolate as robust, lateralized, time-locked potentials in scalp EEG. Rather, they appear as weak and spatially confined signals that may only become detectable under specific recording conditions or analysis strategies. This interpretation is consistent with methodological considerations emphasizing that sCEPs are often poorly phase-locked and may therefore be only partially captured by conventional time-domain averaging.

### 4.2. Time–Frequency Analysis of Cerebellar Oscillatory Activity

In contrast to time-domain analysis, time–frequency analysis yielded more robust and consistent results. A significant increase in oscillatory power was observed in cerebellar recordings across all comparisons, showing clear side specificity and limited contamination from other sources. For upper-limb stimulation, this increase was most prominent at ~20 Hz, whereas for lower-limb stimulation it was centered at lower frequencies, around ~10 Hz. In both conditions, power increases emerged approximately 50 ms after stimulation, persisted beyond 300 ms and were consistently stronger in CB2–Fz than in all other derivations.

Oscillatory activity is a fundamental property of cerebellar circuits and arises from the interaction of multiple afferent and intrinsic mechanisms operating at distinct temporal scales. Somatosensory input reaches the cerebellar cortex predominantly through mossy fibers, which terminate on granule cells and excite Purkinje cells via parallel fibers, while a second powerful afferent system is provided by climbing fibers originating in the inferior olive [48,49]. These two systems exhibit distinct temporal dynamics that shape cerebellar population activity: mossy fiber–granule cell–parallel fiber transmission supports relatively fast and sustained excitation across extended cortical regions, whereas climbing fiber activation produces strong, synchronous depolarization of Purkinje cell somata and proximal dendrites [50,51]. Importantly, the inferior olive possesses intrinsic subthreshold oscillatory properties, most prominently in the 7–10 Hz range, and olivary neurons are electrically coupled via gap junctions, enabling synchronized oscillations across neuronal clusters [52,53]. These oscillations can entrain climbing fiber discharge and impose rhythmic structure on cerebellar cortical activity, particularly within the theta–alpha frequency range. In addition to olivocerebellar rhythmicity, oscillatory phenomena have been described within the cerebellar cortex itself, especially in the granule cell layer, where local field potential oscillations spanning approximately 4–25 Hz depend on the spatial organization of mossy fiber input and Golgi cell inhibition [54,55]. These oscillations exhibit a modular, often parasagittal organization and can dynamically synchronize across cerebellar regions during sensorimotor tasks, supporting the spatiotemporal coordination of information flow [56]. At the network level, alpha–beta oscillations provide repeated windows of increased excitability lasting tens of milliseconds, which are well matched to the relatively slow conduction velocity of parallel fibers and may facilitate coherent activation of distributed Purkinje cell populations.

In humans, MEG studies have demonstrated that somatosensory stimulation elicits stimulus-locked cerebellar oscillations at frequencies closely matching the physiological properties of cerebellar afferent systems. Tesche and Karhu reported cerebellar oscillations in the 6–12 Hz and 25–35 Hz ranges following median nerve stimulation, interpreted as population-level signatures of climbing fiber- and mossy fiber-mediated activity, respectively [40]. These oscillations were sustained during stimulus omission, exhibited anticipatory enhancement prior to expected stimuli, and were strongly modulated by temporal predictability and attentional state. Moreover, cerebellar oscillatory responses showed refractory properties distinct from those observed in primary somatosensory cortex, indicating different temporal dynamics for maintaining neuronal representations in cerebellar versus cortical networks [40].

Taken together, prior invasive and non-invasive studies converge in showing that somatosensory stimulation reliably engages intrinsic cerebellar oscillatory networks, providing a physiological framework for interpreting the oscillatory responses observed in the present EEG recordings as genuine cerebellar activity rather than nonspecific volume-conducted signals [57]. Within this framework, the limb-dependent frequency shift observed in the present study can be interpreted as the interaction between intrinsic cerebellar oscillatory mechanisms and differences in the temporal structure of afferent input. In addition to differences in conduction distance, alternative factors may contribute to this effect, including differences in afferent synchronization, temporal dispersion of the sensory volley, scaling of sensorimotor representations, and peripheral or stimulus-related factors. Compared with median nerve stimulation, tibial nerve stimulation involves longer conduction distances and greater temporal dispersion of the somatosensory volley, resulting in reduced phase coherence and attenuation of higher-frequency components. This temporal dispersion biases cerebellar responses toward lower-frequency oscillations, consistent with the ~10 Hz power increase observed for lower-limb stimulation. In contrast, upper-limb afferents reach the cerebellum with shorter latencies and greater temporal precision, favoring synchronization at higher frequencies and resulting in a dominant oscillatory response around ~20 Hz. Overall, this pattern supports the view that cerebellar engagement in somatosensory processing is more faithfully captured in the time–frequency domain than by conventional time-domain averaging, and that oscillatory dynamics provide a sensitive and physiologically grounded marker of cerebellar contributions to temporally structured sensory processing. From a translational perspective, cerebellar oscillatory measures may represent promising candidate biomarkers for monitoring cerebellar function in spinocerebellar disorders. In future studies, such measures could be integrated within intensive motor training and rehabilitation protocols to objectively track cerebellar engagement and treatment-related plasticity, as suggested by recent work on cerebellar rehabilitation [58].

## 5. Limitations and Future Directions

The present study has several limitations that should be acknowledged. The sample size, albeit in line with previous work recording cerebellar activity non-invasively, is limited, and interindividual variability of responses has not been addressed. Posterior fossa EEG recordings remain susceptible to volume conduction and contributions from non-cerebellar generators, including brainstem nuclei and deep cerebellar nuclei, and the spatial specificity of scalp EEG is inherently limited, particularly in the time domain. Although lateralization in the bipolar CB2–CB1 derivation and frequency-specific oscillatory responses provide stronger support for cerebellar involvement, they do not allow complete exclusion of subcortical sources, even in the time–frequency domain. Accordingly, the present results should be interpreted as evidence for cerebellar engagement rather than as proof of exclusive cerebellar generation. In addition, frequency-specific effects were identified in an exploratory manner and should be confirmed in independent datasets. Future studies using techniques such as MEG or source-informed analyses will be important to further disambiguate cerebellar from subcortical contributions. Longitudinal and interventional designs, particularly in clinical populations, will also be needed to determine the sensitivity of cerebellar oscillatory markers to disease progression and training-induced plasticity.

## 6. Conclusions

The present findings demonstrate that sCEPs to peripheral nerve stimulation can be detected non-invasively using scalp EEG, particularly when analyzed in the time–frequency domain. While time-domain analyses are strongly constrained by reference effects and subcortical contamination, oscillatory responses provide a more robust and spatially specific marker of cerebellar involvement. The ability to capture cerebellar oscillatory activity following both upper- and lower-limb stimulation opens new perspectives for investigating cerebellar physiology and its role in somatosensory processing. Importantly, this approach may represent a promising avenue for future investigation in conditions affecting the lateral portions of the spinal cord, where spinocerebellar pathways are primarily located and may be compromised despite preserved dorsal column function. In this context, cerebellar electrophysiological measures could, following further validation, complement conventional SEPs and contribute to the assessment of spinocerebellar pathway integrity and cerebellar function in both health and disease.

## Figures and Tables

**Table 1:** Electrode montage. APBa: active electrode for the abductor pollicis brevis muscle; APBr: reference electrode for the abductor pollicis brevis muscle; AHa: active electrode for the abductor hallucis; AHr: reference electrode for the abductor hallucis. See text for details.

Upper-limb (median nerve) SEPs		
CP3	Fz	Cortical component N20/P25
Fz	IpsS	Bulbar component P14/18
C5s	Jn	Spinal cord N13
CB1	Fz	Cerebellar SEPs (contralateral)
CB2	Fz	Cerebellar SEPs (ipsilateral)
SP2	Fz	Electromyographic activity from the neck
APBa	APBr	Compound muscle action potential from APB
Lower-limb (tibial nerve) SEPs		
CPz	Fz	Cortical component P40/N45
Fz	C5s	Bulbar component P31/N34
Oz	Fz	Additional posterior fossa channel
CB1	Fz	Cerebellar SEPs (contralateral)
CB2	Fz	Cerebellar SEPs (ipsilateral)
SP2	Fz	Electromyographic activity from the neck
AHa	AHr	Compound muscle action potential from AH

## Data Availability

The data presented in this study are available upon request from the corresponding author. The data are not publicly available due to privacy restrictions.

## Abbreviations

The following abbreviations are used in this manuscript:

ECeG	Electrocerebellogram
SEPs	Somatosensory evoked potentials
CMAP	Compound muscle action potential
sCEPs	Cerebellar evoked potentials (sCEPs)
